# Inpatient to Outpatient Shifts in Surgical Care: Persistence of COVID-19 Era Changes and Socioeconomic Variations

**DOI:** 10.1177/10775587251396718

**Published:** 2025-12-06

**Authors:** Angela T. Chen, Philip A. Saynisch, Hummy Song, Aaron Smith-McLallen, Guy David, Alon Bergman

**Affiliations:** 1 University of Pennsylvania, Philadelphia, USA; 2 Independence Blue Cross, Philadelphia, PA, USA

**Keywords:** outpatient setting, COVID-19, elective surgery, health care utilization, disparities

## Abstract

The COVID-19 pandemic disrupted surgical care delivery, yet the extent to which shifts from inpatient to outpatient settings have persisted remains unclear. Using medical claims data from Independence Blue Cross (2018–2022), we examined changes in surgery settings across 102 procedures before the pandemic and during the 2 years following the suspension of elective surgeries. After 2 years, inpatient volumes decreased for 9 of the 20 most common pre-pandemic inpatient procedures, with corresponding increases in outpatient utilization. Hip and knee replacements experienced the most pronounced shifts, with inpatient shares falling by more than 40 percentage points. Patients from lower-income census tracts saw greater declines in overall procedure volumes (–6.0%) compared to those from higher-income areas (+5.2%). Total allowed amounts decreased for procedures with outpatient migration, while out-of-pocket costs remained stable. These findings suggest durable, post-pandemic shifts in surgical care delivery patterns, with potential implications for access, costs, and equity.

## Introduction

Days after the U.S. president declared the COVID-19 pandemic a national emergency, the Centers for Medicare and Medicaid Services (CMS) suspended all elective surgeries to prioritize resources for inpatient infection control and surge capacity (CMS, 2020b; Dobbs et al., 2023; FEMA, 2020). This measure resulted in a 1-month halt to virtually all non-emergent surgical care delivery (CMS, 2020a), which has since prompted considerable research interest. Utilization patterns have revealed a significant drop in visits for elective surgeries, with many procedures failing to return to pre-pandemic levels by 2021 (Ghoshal et al., 2022; Mattingly et al., 2021). This decline had profound financial implications for health care institutions (Bose et al., 2021; Mazzaferro et al., 2022; Tonna et al., 2020; Waitzberg et al., 2021) and delayed planned clinical care for patients (Lee et al., 2022; Song et al., 2021).

While existing work has documented the immediate effects of elective surgery suspensions, pandemic-era changes in the balance between inpatient and outpatient settings have received limited attention. Early evidence indicates a shift toward outpatient surgeries (Shao et al., 2022; Shariq et al., 2023), likely driven by efforts to reduce inpatient burden. However, it remains unclear whether surgical care settings have reverted to pre-pandemic patterns or if a shift toward outpatient settings has persisted. Moreover, little is known about how these trends vary across demographic groups.

The distribution of procedures between inpatient and outpatient settings has significant implications for health care delivery. Outpatient settings for appropriate patients offer cost-effective alternatives to inpatient care (Castells et al., 2001; Crawford et al., 2015), may increase surgical capacity (Jain et al., 2020), and can improve the patient experience (Kelly et al., 2018). Five years after the pandemic began, we are now able to observe whether changes in care delivery have persisted. In this study, we analyze data from Independence Blue Cross (IBX), a large private health insurer in southeastern Pennsylvania, to describe changes in the site of care for elective procedures during the first 2 years after elective surgeries resumed. We document the persistence of shifts from inpatient to outpatient settings and examine whether these shifts vary by procedure type and patient demographics, providing insight into the evolving landscape of surgical care delivery.

## New Contribution

This study contributes to the growing literature on how the COVID-19 pandemic influenced health care delivery by providing a 2-year post-pandemic analysis of changes in surgical care settings. While prior research has focused on the immediate disruption to surgical volumes following the suspension of elective procedures (Ghoshal et al., 2022; Mattingly et al., 2021; Shao et al., 2022; Shariq et al., 2023), we extend this work by examining the persistence of shifts toward outpatient care across multiple high-volume procedures and by assessing how these trends varied across sociodemographic groups.

## Study Data and Methods

### Data and Study Population

In this observational study, we analyzed medical claims data from IBX spanning from 2018 to 2022, with date elements at the week level. We defined three distinct periods: the pre-pandemic period (“Pre-”) from Week 1 of 2018 through Week 10 of 2020 (approximately January 1, 2018 to March 10, 2020); the first year post-suspension of elective surgeries (“Post1”) from Week 20 of 2020 through Week 19 of 2021 (approximately May 11, 2020 to May 15, 2021); and the second year post-suspension (“Post2”) from Week 20 of 2021 through Week 19 of 2022 (approximately May 16, 2021 to May 14, 2022).

Our study population included all patients who underwent any procedure during the study period in either inpatient or outpatient settings. The site of care for each claim was determined using standard place of service codes (CMS, 2023). Outpatient procedures included those at both hospital-based outpatient centers and ambulatory surgical centers. We grouped procedures by Current Procedural Terminology (CPT) codes into clinically meaningful categories using the Clinical Classifications Software (CCS) (HCUP, 2021) developed by the Agency for Healthcare Research and Quality.

### Measures and Analyses

Our primary outcome measures were inpatient procedure volume and inpatient procedure share. To account for changes in plan enrollment over time, we expressed procedure volumes as the number of surgeries per week per one million enrollees, calculated separately for each of the three study periods. Secondary outcomes included the total allowed amount (CMS, 2022) of a surgical visit, total patient out-of-pocket (OOP) costs, and number of 6-month hospital-free days (HFDs) (Auriemma et al., 2021). The total allowed amount was calculated by aggregating allowed amounts on all claims within a 2-week period following a patient’s surgery date. OOP costs were determined by summing all copays, coinsurance amounts, and deductibles on patient claims occurring 2 weeks after the surgery date. HFDs represent the count of days (out of 180) during which a patient was alive, was not admitted, and did not visit the emergency department within 6 months post-surgery.

In our primary analysis, we calculated weekly inpatient procedure volumes per one million enrollees for each CCS procedure group during the three periods and computed the inpatient share of procedures within each group for each period. We also conducted subgroup analyses based on patient demographics including age and census tract-level measures of race, income, and labor force participation. To focus these analyses on procedures with the most significant shifts in care settings, we selected the top five procedures with the largest decreases in the share of inpatient procedures during the study period.

Patient age was obtained from IBX member enrollment records, while data on income, race, and labor force participation were acquired by linking the census tract of residence to information from the U.S. Census Bureau’s 2018 American Community Survey 5-year aggregate census tract-level files. We generated categorical variables for each demographic subgroup by dividing the sample of procedures into quartiles.

For each demographic subgroup, we calculated the percentage change in weekly total volume from the pre-pandemic period baseline to both post-suspension years and determined the percentage point difference in inpatient share. Confidence intervals for the changes in weekly total volume were calculated using the methodology described in the Appendix of Birkmeyer et al. (2020), and confidence intervals for the difference in proportions compared the pre-pandemic and post-pandemic inpatient shares.

To explore how changes in place of service might affect cost and clinical outcomes, we focused on the five procedures with the most substantial changes in care settings. We plotted trends in total allowed amounts, patient OOP costs, and 6-month HFDs by calculating the average annual values for each outcome from 2018 to 2022, stratified by place of service and procedure type, with all monetary values adjusted to 2021 dollars. We also calculated these measures as ratios comparing the highest and lowest quartiles of demographic variables to illustrate trends in relative changes across subgroups.

Data were analyzed in R version 4.2.1 (R Foundation for Statistical Computing), and we adhered to the Strengthening the Reporting of Observational Studies in Epidemiology (STROBE) guidelines. Since the analyses utilized de-identified observational data without personal health information, this study did not qualify as human subjects’ research and was exempt from Institutional Review Board (IRB) review.

## Results

**Figure 1.**
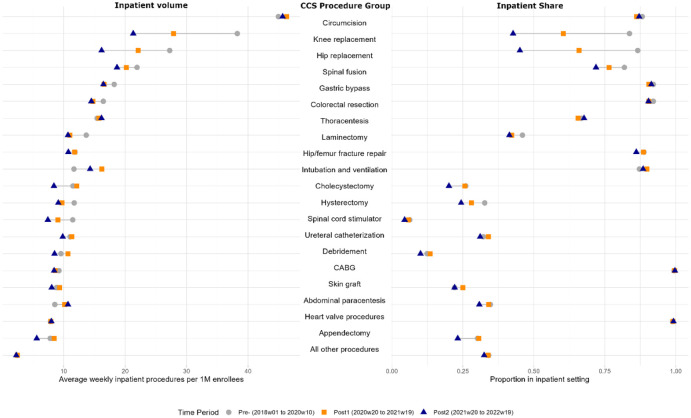
Changes in Inpatient Procedure Volume and Inpatient Procedure Share, Before and After the COVID-19 Elective Surgery Suspension. Data include 771,037 procedures across 102 Clinical Classifications Software (CCS) procedure groups. The top 20 CCS procedure groups with the highest inpatient volume are listed individually on the y-axis, sorted from highest to lowest pre-pandemic weekly inpatient volume per one million enrollees; the remaining 82 procedures are aggregated. The left panel displays changes in inpatient volume, and the right panel shows changes in inpatient share across the three time periods. Time periods are defined as follows: the pre-pandemic period (Week 1 of 2018 through Week 10 of 2020) corresponds to January 1, 2018 through March 10, 2020; “Post1” (Week 20 of 2020 through Week 19 of 2021) corresponds to May 11, 2020 through May 15, 2021; and “Post2” (Week 20 of 2021 through Week 19 of 2022) corresponds to May 16, 2021 through May 14, 2022. Abbreviation: CABG = coronary artery bypass graft.

Figure 1 illustrates inpatient weekly volume and inpatient procedure share in the left and right panels, respectively. Of 771,037 procedures analyzed across 102 CCS procedure groups, the top 20 groups are individually listed by pre-pandemic inpatient weekly volume, while the average of the remaining 82 CCS procedure groups is presented in aggregate. The study sample had a mean age of 51 years (*SD* = 21), with 9.9% of patients under 18 and 2.6% under 1 year of age. Prior to the pandemic, circumcisions were the most commonly performed inpatient procedure, with an average of 44.9 per week per one million enrollees (Supplemental Appendix Table 1), followed by knee replacements (38.3) and hip replacements (27.3). Nine of the top 20 procedures experienced sustained reductions in weekly volume, defined as lower volumes in both post-suspension periods relative to the pre-pandemic baseline. The average decline across all 20 procedures was 12.5%.

The right panel of Figure 1 displays corresponding inpatient shares for these procedures. Coronary artery bypass grafts (CABG) and heart valve procedures maintained about 100% of their volume in the inpatient setting across all time periods (Supplemental Appendix Table 2). Among the top 20 procedures, 13 experienced sustained shifts from inpatient to outpatient settings, with lower inpatient shares and higher outpatient shares in both years following the elective surgery suspension. Hip and knee replacements saw the most significant shifts to outpatient settings, with inpatient proportions decreasing by 0.42 and 0.41, respectively. On average, the inpatient proportion among the top 20 procedures decreased by 0.07.

**Figure 2.**
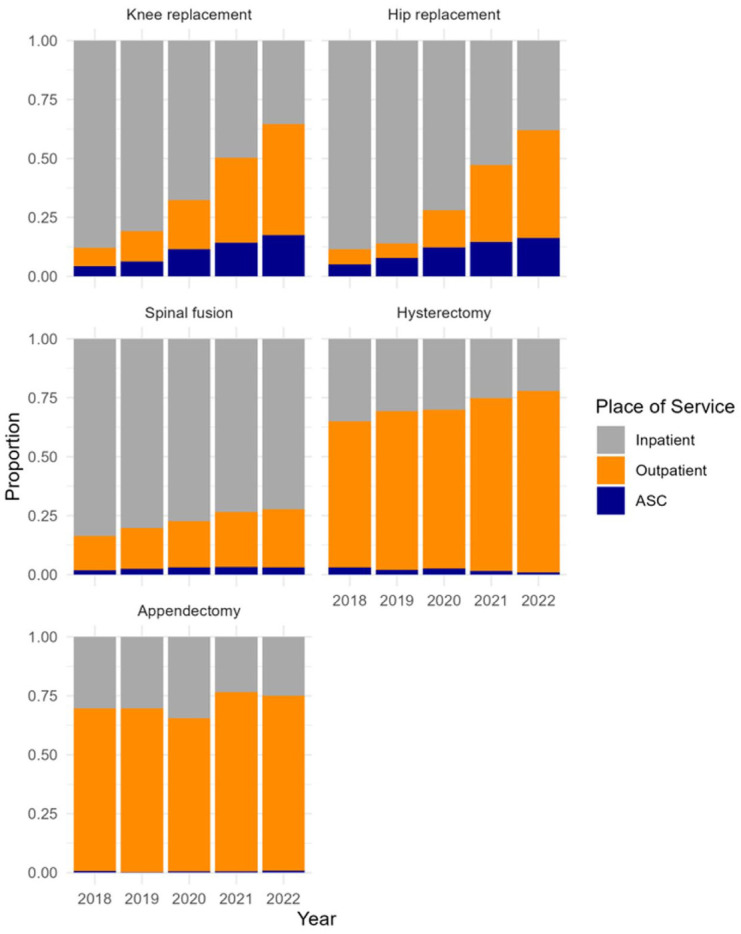
Place of Service Share by Year and Procedure, Top 5 Procedures With the Greatest Change in Inpatient Share From 2018 to 2022. Data include 60,878 procedures across five Clinical Classifications Software (CCS) procedure groups with the greatest change in place of service from the week of January 1, 2018, to the week of May 14, 2022. Procedures are ordered from greatest to least change. In this figure, “outpatient” refers specifically to hospital-based outpatient procedures. Abbreviation: ASC = ambulatory surgery center.

Figure 2 highlights the five procedure groups with the greatest degree of change in inpatient share from the pre-pandemic period to 2 years after the suspension of elective procedures, encompassing 60,878 individual cases. Among these, knee replacement, hip replacement, spinal fusion, and hysterectomy demonstrated a consistent, year-over-year decrease in inpatient share, accompanied by a corresponding rise in outpatient procedures. The growing role of ambulatory surgery centers (ASCs) is also evident, with these facilities performing over 15% of knee and hip replacements by 2022.

### Results by Procedure and Patient Subgroup

Table 1 presents changes in total weekly procedure volume and the proportion performed in inpatient settings, stratified by procedure type and patient demographics, for the five procedures shown in Figure 2. Corresponding confidence intervals are reported in Supplemental Appendix Table 3. In the first year following the suspension, total weekly procedure volume remained largely unchanged from pre-pandemic levels (−0.3%; 95% CI = [−4.3, 3.2]), but the inpatient share decreased by 11.8 percentage points (95% CI = [−12.8, −10.8]). By the second year, weekly volume exceeded pre-COVID levels by 2.9% (95% CI = [2.4, 3.5]), while the inpatient share continued to fall, decreasing by 23.8 percentage points (95% CI = [−24.8, −22.8]) from a baseline of 65.0%. Knee replacements, hysterectomies, and hip replacements—the three most common procedures by volume—all surpassed pre-pandemic volumes by Year 2. In contrast, spinal fusions and appendectomies remained below baseline. Despite this variation in volume recovery, all five procedures saw reductions in the inpatient share, ranging from a 7.0 percentage point drop for appendectomies to a 41.5 percentage point drop for hip replacements.

**Table 1.** Two-Year Changes in Total Procedure Volume and Inpatient Procedure Share Relative to the Pre-Pandemic Baseline, by Subgroup.

Patient subgroups	No. of observations	Weekly volume (all settings) per 1M enrollees			Percent inpatient		
		Baseline	Change from baseline, %^a^		Baseline	Change from baseline, percentage point difference	
		Pre-	Year 1 post	Year 2 post	Pre-	Year 1 post	Year 2 post
*Procedures* ^b^	60,878	159.6	–0.3	2.9	65.0	–11.8	–23.8
Knee replacement	17,041	45.5	–0.2	7.7	83.7	–23.3	–41.0
Hysterectomy	12,739	34.5	–4.1	0.9	32.7	–4.7	–8.3
Hip replacement	12,030	31.4	4.8	9.6	86.6	–20.7	–41.5
Spinal fusion	9,901	26.7	–1.1	–3.4	81.9	–5.4	–10.0
Appendectomy	9,167	24.4	–0.8	–6.6	30.2	0.3	–7.0
*Age*							
Oldest quartile (≥64 years)	15,946	169.6	–3.8	9.1	86.6	–14.8	–33.1
Middle quartiles	30,102	159.0	0.9	2.0	68.3	–14.0	–26.9
Youngest quartile (<46 years)	14,830	156.7	1.2	–2.0	35.1	–3.1	–9.1
*Race, % Black/African American* ^c^							
Highest quartile (≥13%)	15,039	164.6	–5.6	–2.3	67.2	–9.5	–23.0
Middle quartiles	29,162	153.5	–0.8	2.6	65.5	–11.8	–23.7
Lowest quartile (<1.5%)	15,087	160.3	0.4	–0.1	62.2	–13.3	–23.9
*Income*							
Highest quartile (≥101K)	14,629	152.9	3.2	5.2	64.8	–12.1	–22.6
Middle quartiles	29,872	158.9	–3.2	1.7	64.6	–11.7	–24.6
Lowest quartile (<57K)	14,785	161.7	–4.5	–6.0	66.5	–10.8	–22.1
*Labor Force Participation Rate*							
Highest quartile (≥71%)	14,901	156.3	0.8	4.6	63.7	–11.7	–24.2
Middle quartiles	29,392	156.3	–1.9	0.6	64.9	–11.6	–23.3
Lowest quartile (<62%)	14,994	162.9	–4.6	–3.3	67.0	–11.6	–23.3

a Detailed reporting of corresponding confidence intervals is included in the Supplemental Appendix. All changes are reported relative to the baseline values.

b Knee replacement, hysterectomy, hip replacement, spinal fusion, and appendectomy represent the five Clinical Classifications Software (CCS) procedure groups with the greatest change in place of service from the week of January 1, 2018, to the week of May 14, 2022.

c Data on race, income, and labor force participation were acquired by linking patients’ census tract of residence to information from the U.S. Census Bureau’s 2018 American Community Survey 5-year aggregate Census tract-level files. Race, income, and labor force participation rates are reported at the census tract level. Demographic data were unavailable for a small portion of census tracts, resulting in a 2.6% missingness rate for race, income, and labor force participation rates.

In both years following the suspension of elective surgeries, changes in surgical volume varied by patient demographics. Patients ages 64 and older experienced a 3.8% decrease in procedure volume in the first year following the suspension of elective surgeries (95% CI = [−4.2, −3.5]), followed by a 9.1% increase (95% CI = [8.8, 9.5]) in the second year, relative to the pre-pandemic baseline. This group also saw substantial shifts toward outpatient care, with a 33.1 percentage point decrease (95% CI = [−34.1, −32.1]) in the inpatient share. In contrast, patients in the youngest age quartile saw a more modest 1.9% decrease in volume (95% CI = [−2.3, −1.7]) and a smaller reduction of 9.1 percentage points in inpatient share (95% CI = [−10.1, −8.1]) over the same period.

Patients residing in census tracts in the highest quartile of Black population share saw a 2.3% reduction (95% CI = [−2.7, −2.0]) in weekly procedure volume 2 years after the pandemic began. Sustained reductions in volume were also most pronounced among patients from census tracts with the lowest median income (−6.0%; 95% CI = [−6.3, −5.7]) and lowest labor force participation (−3.3%; 95% CI = [−3.5, −3.0]). By comparison, patients living in areas with the highest median income experienced a 5.2% increase in volume (95% CI = [4.9, 5.5]), and those in areas with the highest labor force participation saw a 4.6% increase (95% CI = [4.3, 5.0]).

Differences in place of service composition across demographic groups were more subtle. The change in the inpatient share of procedures was comparable across all demographic subgroups not based on age, ranging from a 24.6 percentage point decrease among patients from census tracts in the middle-income quartiles (95% CI = [−28.9, −20.4]) to a 22.1 percentage point decrease among patients in the lowest income quartile (95% CI = [−24.1, −20.1]).

### Financial and Clinical Outcomes

Inpatient procedures had allowed amounts approximately double that of outpatient procedures (Supplemental Appendix Figure 1). Patients receiving inpatient care had slightly lower 6-month HFDs compared to those treated as outpatients, while OOP costs were similar between the two groups. Between 2018 and 2022, costs (both allowed amounts and OOP costs) for both inpatient and outpatient visits remained relatively constant. There was a subtle downward trend in HFDs for inpatient visits, whereas outpatient HFDs remained relatively stable across all years.

Among the five procedures, all except appendectomy saw a decrease in the total allowed amount. Knee and hip replacements started at peak levels in 2018, at $32,075 (*SE* = $277) and $31,562 (*SE* = $374; Supplemental Appendix Table 4), respectively, and decreased to an average allowed amount of $24,686 (*SE* = $380) and $25,172 (*SE* = $500) by 2022. Spinal fusions demonstrated the largest change in allowed amount, decreasing from an average of $65,946 (*SE* = $1,064) in 2018 to $58,751 (*SE* = $1,899) in 2022. Allowed amounts for appendectomies, which exhibited the smallest outpatient shift, remained stable.

Trends in OOP costs were less clear. OOP costs for knee replacements, hip replacements, and hysterectomies stayed relatively constant, while they decreased slightly for appendectomy (from $1,473 in 2018 [*SE* = $40.7] to $1,344 in 2022 [*SE* = $72.8]) and increased slightly for spinal fusion (from $1,346 in 2018 [*SE* = $37.5] to $1,511 in 2022 [*SE* = $73.6]). In addition, there was a minor increase in HFDs over time for joint replacements and hysterectomies, although the change was minimal, amounting to less than 1 day. No major changes were observed in relative costs or clinical outcomes between demographic subgroups (Supplemental Appendix Figure 2).

## Discussion

The COVID-19 pandemic era coincided with notable shifts in surgical care delivery. Among the 20 highest-volume inpatient procedures in the pre-pandemic period, nearly half experienced sustained reductions in inpatient volume 2 years after the suspension of elective surgeries, accompanied by increases in the share performed in outpatient settings. While initial shifts likely reflected pragmatic efforts to preserve inpatient capacity during the acute phase of the pandemic, these patterns appear to have persisted, suggesting a potential longer-term realignment in the setting of elective surgical care.

The most pronounced shifts were observed in joint replacements, where the inpatient share decreased by more than 40 percentage points. This trend builds on the pre-pandemic movement toward outpatient arthroplasty (Piple et al., 2023), catalyzed in part by CMS’s removal of the inpatient-only designation for total knee and hip replacements (Iorio et al., 2020; Krueger et al., 2020). The continued—and even accelerated—reduction in inpatient share during the pandemic period likely reflects a combination of these ongoing policy changes and evolving preferences among providers and patients. Similar patterns were observed for other procedures, including case-appropriate hysterectomies (Cappuccio et al., 2021; Moawad et al., 2017) and appendectomies (Frazee et al., 2014). In contrast, categories such as newborn circumcisions and CABGs illustrate the relative inelasticity of birth-related and life-saving inpatient services, which remained largely stable despite pandemic disruptions.

Our analysis also identified age-related variation in surgical care delivery patterns. Among adults aged 64 and older, procedure volumes initially decreased but rebounded in the second year, possibly reflecting greater caution in seeking care during the early pandemic due to heightened COVID-19 risk (Berlin et al., 2024), followed by a return in demand as concerns subsided. Older adults also experienced a larger shift toward outpatient settings compared to the youngest age quartile, which could reflect a combination of infection-related concerns associated with inpatient care and a higher prevalence of conditions—such as joint disease—that are increasingly managed in outpatient settings.

Socioeconomic variation in total procedure volume raises important concerns. Among the five procedures with the most pronounced shifts to outpatient care, patients residing in census tracts in the lowest income quartile experienced a 6.0% decrease in total volume relative to pre-pandemic levels, while those in the highest quartile saw a 5.2% increase. Similar disparities emerged by labor force participation and, to a lesser extent, by census tract racial composition. Although prior research suggests the pandemic did not worsen disparities in access to major inpatient surgeries (Glance et al., 2022), our findings point to potential emerging barriers to elective outpatient care for socioeconomically disadvantaged populations. Pandemic-related requirements, such as preoperative COVID-19 screening, may have contributed to delays in care by imposing disproportionate burdens on resource-limited individuals (Lin et al., 2023).

While disparities in overall procedure volume were evident, the magnitude of the shift from inpatient to outpatient settings was relatively consistent across socioeconomic subgroups. Most groups experienced similar reductions in inpatient share—generally between 22 and 24 percentage points. This uniformity, however, should not be interpreted as the absence of disparities. Prior research indicates that patients from areas with higher proportions of Black residents, lower incomes, and lower labor force participation have historically relied more heavily on inpatient care (Chatterjee et al., 2022; Strope et al., 2009). As a result, even with similar shifts across groups, pre-existing differences in access to outpatient surgical settings persist and merit continued policy and research attention. Future research should identify the patients whose health care utilization patterns are most sensitive to changes in the cost or availability of services and evaluate the long-term health and economic consequences of foregone procedures in these populations. Health systems may also need to refine provider incentives, such as through improved risk adjustment, to ensure that efficiency gains from outpatient migration do not come at the expense of access for patients with the greatest medical need.

The shift toward outpatient settings was generally associated with reductions in total procedure costs, as measured by allowed amounts. Procedures such as joint replacements and spinal fusions—those with the most substantial migration to outpatient care—also experienced the largest decreases in average allowed amounts, likely reflecting lower costs of care delivered in outpatient versus inpatient environments. Notably, however, patient OOP spending remained largely unchanged despite the overall cost reductions. This disconnect highlights an important policy consideration: system-level cost savings do not necessarily translate into financial relief for patients. The persistence of stable OOP expenses warrants closer examination, particularly regarding its impact on financially vulnerable populations.

Several limitations should be considered when interpreting these findings. First, the analysis is based on claims data from a single commercial insurer in southeastern Pennsylvania, which limits generalizability to other regions, insurance markets, and populations with different demographic and health care characteristics. These findings may not reflect patterns in areas with distinct care infrastructures or among publicly insured or uninsured populations.^1^ In addition, our data end in mid-2022, limiting our ability to assess the persistence of these care delivery patterns in subsequent years. The data set excludes Medicaid beneficiaries, traditional Medicare enrollees, and uninsured individuals—groups that may have experienced different care disruptions and face unique barriers to outpatient surgical care. These exclusions may limit our ability to fully assess disparities, particularly among socioeconomically disadvantaged or racially diverse populations. Although we adjust for changes in enrollment over time, we cannot fully account for selective disenrollment or insurance switching, which may differ by socioeconomic status. Furthermore, our use of census tract-level socioeconomic indicators provides only proxy measures of individual characteristics, potentially masking important within-tract heterogeneity. Finally, this is a descriptive study. We cannot attribute observed trends solely to the pandemic, as other contemporaneous factors—such as policy changes, technology adoption, and evolving preferences—likely also play a role. Future work using multi-payer data and more granular individual-level measures would further strengthen understanding of these trends.

Our findings document a sustained shift in surgical care delivery during the COVID-19 era, marked by declining inpatient volumes and increased use of outpatient settings for common elective procedures. Initially driven by the need to preserve inpatient capacity, these patterns now suggest a longer-term realignment. The increase in outpatient care can offer potential benefits, such as increased efficiency and reduced costs. However, disparities in procedure volumes among socioeconomically disadvantaged populations raise concerns about equitable access. Furthermore, the absence of corresponding reductions in patient OOP spending, despite lower total allowed amounts, highlights a disconnect between system-level savings and patient financial burden. As surgical care continues to evolve, policymakers and health system leaders must monitor the implications for access, quality, and equity. Future research should explore the long-term effects of these shifts, with particular attention to ensuring that the benefits of outpatient migration extend across all patient populations.

## Supplemental Material

sj-docx-1-mcr-10.1177_10775587251396718 – Supplemental material for Inpatient to Outpatient Shifts in Surgical Care: Persistence of COVID-19 Era Changes and Socioeconomic Variations Supplemental material, sj-docx-1-mcr-10.1177_10775587251396718 for Inpatient to Outpatient Shifts in Surgical Care: Persistence of COVID-19 Era Changes and Socioeconomic Variations by Angela T. Chen, Philip A. Saynisch, Hummy Song, Aaron Smith-McLallen, Guy David and Alon Bergman in Medical Care Research and Review

## Notes

1. While we benchmarked our results against the HCUP National Inpatient Sample (NIS), direct comparisons are limited because (a) our study periods are defined weekly, whereas NIS can only be queried by calendar year, and (b) NIS classifies procedures using ICD-10-PCS codes for inpatient discharges, while our study uses CPT codes that capture procedures across both inpatient and outpatient settings. Some divergence is therefore expected. Nonetheless, the most common inpatient procedures in our study (including knee replacement, hip replacement, spine fusion, hysterectomy, and appendectomy) also rank among the most frequent in NIS, and both sources document declines in inpatient volumes.
