# Supplementary material for: Inpatient to Outpatient Shifts in Surgical Care: Persistence of COVID-19 Era Changes and Socioeconomic Variations
Source: Med Care Res Rev. 2025 Dec 6;83(2):156–64. doi: 10.1177/10775587251396718 (PMC12946234; doi:10.1177/10775587251396718)
Supplement: sj-docx-1-mcr-10.1177_10775587251396718 – Supplemental material for Inpatient to Outpatient Shifts in Surgical Care: Persistence of COVID-19 Era Changes and Socioeconomic Variations [file sj-docx-1-mcr-10.1177_10775587251396718.docx]

**Appendix Exhibits**

**Appendix Table 1. Detailed reporting of inpatient weekly volume, before and after the COVID-19 elective surgery suspension**

| **Procedure**^a^ | **Total inpatient volume, pre-pandemic** | **Average weekly inpatient volume per 1 million enrollees** | | | **% change, Pre- to Post2** |
| --- | --- | --- | --- | --- | --- |
|  |  | **Pre-** | **Post1** | **Post2** |  |
| Circumcision | 8981 | 44.9 | 46.3 | 45.6 | 1.6% |
| Knee replacement | 7362 | 38.3 | 27.9 | 21.3 | -44% |
| Hip replacement | 5274 | 27.3 | 22.1 | 16.2 | -41% |
| Spinal fusion | 4265 | 21.9 | 20.2 | 18.7 | -15% |
| Gastric bypass | 3430 | 18.2 | 16.6 | 16.5 | -9.3% |
| Colorectal resection | 3188 | 16.4 | 14.7 | 14.5 | -12% |
| Thoracentesis | 3018 | 15.4 | 15.8 | 16.1 | 4.5% |
| Laminectomy | 2579 | 13.7 | 11.0 | 10.7 | -22% |
| Hip/femur fracture repair | 2286 | 11.8 | 11.8 | 10.8 | -8.5% |
| Intubation and ventilation | 2284 | 11.7 | 16.2 | 14.3 | 22% |
| Cholecystectomy | 2208 | 11.5 | 12.1 | 8.4 | -27% |
| Hysterectomy | 2205 | 11.7 | 9.7 | 9.1 | -22% |
| Spinal cord stimulator | 2125 | 11.5 | 9.0 | 7.4 | -36% |
| Ureteral catheterization | 2110 | 11.0 | 11.3 | 9.8 | -11% |
| Debridement | 1844 | 9.5 | 10.7 | 8.5 | -11% |
| CABG | 1761 | 9.2 | 8.6 | 8.4 | -8.7% |
| Skin graft | 1638 | 8.8 | 9.3 | 8.0 | -9.1% |
| Abdominal paracentesis | 1633 | 8.5 | 10.2 | 10.7 | 26% |
| Heart valve procedures | 1458 | 7.9 | 7.9 | 8.0 | 1.3% |
| Appendectomy | 1453 | 7.8 | 8.4 | 5.6 | -28% |
| All other procedures (n = 82) | 30,817 | 2.4 | 2.5 | 2.3 | -12% |

^a^ Individually-listed procedures listed are the top 20 Clinical Classifications Software (CCS) procedure groups with the highest inpatient volume.

**Appendix Table 2. Detailed reporting of inpatient procedure share, before and after the COVID-19 elective surgery suspension**

| **Procedure^a^** | **Total inpatient volume, pre-pandemic** | **Inpatient share** | | | **Share difference, Pre- to Post2** |
| --- | --- | --- | --- | --- | --- |
|  |  | **Pre-** | **Post1** | **Post2** |  |
| Circumcision | 8981 | 0.88 | 0.86 | 0.87 | -0.01 |
| Knee replacement | 7362 | 0.84 | 0.60 | 0.43 | -0.41 |
| Hip replacement | 5274 | 0.87 | 0.66 | 0.45 | -0.42 |
| Spinal fusion | 4265 | 0.82 | 0.77 | 0.72 | -0.10 |
| Gastric bypass | 3430 | 0.92 | 0.91 | 0.91 | -0.01 |
| Colorectal resection | 3188 | 0.92 | 0.91 | 0.90 | -0.02 |
| Thoracentesis | 3018 | 0.67 | 0.66 | 0.68 | 0.01 |
| Laminectomy | 2579 | 0.46 | 0.42 | 0.41 | -0.05 |
| Hip/femur fracture repair | 2286 | 0.89 | 0.89 | 0.86 | -0.03 |
| Intubation and ventilation | 2284 | 0.87 | 0.90 | 0.89 | 0.01 |
| Cholecystectomy | 2208 | 0.26 | 0.26 | 0.20 | -0.06 |
| Hysterectomy | 2205 | 0.33 | 0.28 | 0.24 | -0.08 |
| Spinal cord stimulator | 2125 | 0.06 | 0.06 | 0.05 | -0.02 |
| Ureteral catheterization | 2110 | 0.32 | 0.34 | 0.31 | -0.01 |
| Debridement | 1844 | 0.12 | 0.14 | 0.10 | -0.02 |
| CABG | 1761 | 0.99 | 1.00 | 1.00 | 0.00 |
| Skin graft | 1638 | 0.22 | 0.25 | 0.22 | 0.00 |
| Abdominal paracentesis | 1633 | 0.35 | 0.34 | 0.31 | -0.04 |
| Heart valve procedures | 1458 | 0.99 | 0.99 | 0.99 | 0.00 |
| Appendectomy | 1453 | 0.30 | 0.31 | 0.23 | -0.07 |
| All other procedures (n = 82) | 30,817 | 0.34 | 0.34 | 0.33 | -0.02 |

^a^ Individually-listed procedures listed are the top 20 Clinical Classifications Software (CCS) procedure groups with the highest inpatient volume.

**Appendix Table 3. Changes in inpatient procedures relative to baseline**

|  |  | ***Weekly total volume, all settings*** | | | ***Percent inpatient*** | | |
| --- | --- | --- | --- | --- | --- | --- | --- |
|  |  | Baseline | Change from baseline, % | | Baseline | Change from baseline,  percentage point difference | |
| Patient subgroups | No. of observations | *Pre-* | *Year 1 Post* | *Year 2 Post* | *Pre-* | *Year 1 Post* | *Year 2 Post* |
| *Procedures*^a^ | 60,878 | 159.6 | -0.31  [-0.88, 0.25] ^b^ | 2.94  [2.36, 3.53] | 65.0 | -11.8  [-12.8, -10.8]^c^ | -23.8  [-24.8, -22.8] |
| Knee replacement | 17,041 | 45.5 | -0.22  [-0.58, 0.14] | 7.69  [7.32, 8.06] | 83.7 | -23.3  [-24.2, -22.4] | -41.0  [-41.9, 40.1] |
| Hysterectomy | 12,739 | 34.5 | -4.06  [-4.36, -3.76] | 0.87  [0.55, 1.19] | 32.7 | -4.67  [-5.58, -3.75] | -8.29  [-9.34, -7.24] |
| Hip replacement | 12,030 | 31.4 | 4.78  [4.46, 5.09] | 9.55  [9.25, 9.86] | 86.6 | -20.7  [-21.6, -19.8] | -41.5  [-42.4, -40.6] |
| Spinal fusion | 9,901 | 26.7 | -1.12  [-1.40, -0.85] | -3.37  [-3.63, -3.11] | 81.9 | -5.44  [-7.9, -3.0] | -9.96  [-12.43, -7.49] |
| Appendectomy | 9,167 | 24.4 | -0.82  [-1.06, -0.58] | -6.56  [-6.79, -6.32] | 30.2 | 0.33  [-0.58, 1.24] | -7.01  [-7.87, -6.15] |
|  |  |  |  |  |  |  |  |
| *Age* |  |  |  |  |  |  |  |
| Oldest quartile (≥64 years) | 15,946 | 169.6 | -3.83  [-4.18, -3.48] | 9.14  [8.79, 9.49] | 86.6 | -14.8  [-15.7, 13.8] | -33.1  [-34.1, -32.1] |
| Middle quartiles | 30,102 | 159.0 | 0.88  [0.44, 1.32] | 1.95  [1.50, 2.40] | 68.3 | -14.0  [-15.0, -13.1] | -26.9  [-27.9, -25.9] |
| Youngest quartile (<46 years) | 14,830 | 156.7 | 1.21  [0.91, 1.51] | -1.98  [-2.27, -1.68] | 35.1 | -3.11  [-4.09, -2.14] | -9.08  [-10.1, -8.11] |
|  |  |  |  |  |  |  |  |
| *Race - % Black/African American*^d^ |  |  |  |  |  |  |  |
| Highest quartile (≥13%) | 15039 | 164.6 | -5.59  [-5.93, -5.25] | -2.34  [-2.68, -2.00] | 67.2 | -9.47  [-11.42, -7.52] | -23.0  [-25.0, -21.1] |
| Middle quartiles | 29162 | 153.5 | -0.78  [-1.18, -0.38] | -2.61  [-2.19, -3.03] | 65.5 | -11.75  [-13.2, -10.3] | -23.7  [-25.1, -22.3] |
| Lowest quartile (<1.5%) | 15087 | 160.3 | 0.43  [0.11, 0.74] | -0.07  [-0.37, 0.22] | 62.2 | -13.3  [-15.2, -11.3] | -23.9  [-25.9, -21.9] |
|  |  |  |  |  |  |  |  |
| *Income* |  |  |  |  |  |  |  |
| Highest quartile (≥$101,000) | 14629 | 152.9 | 3.21  [2.90, 3.53] | 5.18  [4.85, 5.52] | 64.8 | -12.1  [-14.1, -10.1] | -22.6  [-24.9, -20.3] |
| Middle quartiles | 29872 | 158.9 | -3.24  [-3.65, -2.82] | 1.69  [1.29, 2.08] | 64.6 | -11.7  [-13.1, -10.3] | -24.6  [-28.9, -20.4] |
| Lowest quartile (<$57,000) | 14785 | 161.7 | -4.52  [-4.84, -4.20] | -5.99  [-6.29, -5.68] | 66.5 | -10.8  [-12.8, -8.85] | -22.1  [-24.1, -20.1] |
|  |  |  |  |  |  |  |  |
| *Labor Force Participation Rate* |  |  |  |  |  |  |  |
| Highest quartile (≥71%) | 14901 | 156.3 | 0.75  [0.45, 1.05] | 4.62  [4.29, 4.96] | 63.7 | -11.7  [-13.7, -9.76] | -24.2  [-30.0, -18.3] |
| Middle quartiles | 29392 | 156.3 | -1.90  [-2.32, -1.49] | 0.60  [0.18, 1.02] | 64.9 | -11.6  [-13.0, -10.1] | -23.3  [-24.7, -21.9] |
| Lowest quartile (<62%) | 14994 | 162.9 | -4.64  [-4.96, -4.33] | -3.25  [-3.54, -2.96] | 67.0 | -11.6  [-13.5, -9.60] | -23.3  [-25.3, -21.3] |

^a^ Knee replacement, hysterectomy, hip replacement, spinal fusion, and appendectomy represent the five Clinical Classifications Software (CCS) procedure groups with the greatest change in place of service from the week of January 1, 2018, to the week of May 14, 2022.

^b^ Confidence intervals for the changes in weekly total volume were calculated using the methodology described in the Appendix of Birkmeyer et al. (2020).^26^

^c^ Confidence intervals for the difference in proportions was used to compare the pre-pandemic and post-pandemic inpatient shares.

^d^ Data on race, income, and labor force participation were acquired by linking patients’ census tract of residence to information from the US Census Bureau’s 2018 American Community Survey 5-year aggregate Census tract-level files. Race, income, and labor force participation rates are reported at the census tract level. Demographic data were unavailable for a small portion of census tracts, resulting in a 2.6% missingness rate for race, income, and labor force participation rates.

**Appendix Table 4. Costs and hospital-free days by place of service and procedure type, 2018-2022**

| *Data by place of service* | | | | | | | | |
| --- | --- | --- | --- | --- | --- | --- | --- | --- |
| Year | Place of service | # of procedures | Allowed amount (mean) | Allowed amount (SE) | OOP (mean) | OOP (SE) | HFDs (mean) | HFDs (SE) |
| 2018 | Inpatient | 9737 | 40332 | 356 | 1280 | 18.2 | 177 | 0.07 |
| 2018 | Outpatient | 4721 | 17320 | 215 | 1308 | 25.9 | 179 | 0.04 |
| 2019 | Inpatient | 9107 | 40044 | 352 | 1297 | 19.3 | 177 | 0.07 |
| 2019 | Outpatient | 5276 | 18086 | 204 | 1323 | 25.0 | 179 | 0.04 |
| 2020 | Inpatient | 7335 | 41733 | 436 | 1373 | 23.1 | 177 | 0.09 |
| 2020 | Outpatient | 5541 | 20246 | 207 | 1358 | 25.7 | 179 | 0.05 |
| 2021 | Inpatient | 6341 | 42796 | 691 | 1324 | 24.0 | 177 | 0.08 |
| 2021 | Outpatient | 7674 | 20008 | 179 | 1307 | 21.1 | 179 | 0.03 |
| 2022 | Inpatient | 1926 | 40984 | 912 | 1318 | 43.4 | 176 | 0.14 |
| 2022 | Outpatient | 3220 | 19254 | 252 | 1292 | 32.2 | 179 | 0.04 |
| *Data by procedure* | | | | | | | | |
| Year | Procedure | # of procedures | Allowed amount (mean) | Allowed amount (SE) | OOP (mean) | OOP (SE) | HFDs (mean) | HFDs (SE) |
| 2018 | Appendectomy | 2209 | 17414 | 431 | 1473 | 40.7 | 177 | 0.10 |
| 2018 | TKR | 4036 | 32075 | 277 | 1138 | 26.0 | 178 | 0.07 |
| 2018 | THR | 2696 | 31562 | 374 | 1302 | 34.2 | 178 | 0.09 |
| 2018 | Hysterectomy | 3044 | 19181 | 301 | 1297 | 32.8 | 178 | 0.08 |
| 2018 | Spinal fusion | 2473 | 65946 | 1064 | 1346 | 37.5 | 176 | 0.20 |
| 2019 | Appendectomy | 2194 | 16691 | 353 | 1517 | 42.7 | 177 | 0.10 |
| 2019 | TKR | 3906 | 31402 | 272 | 1123 | 26.0 | 178 | 0.07 |
| 2019 | THR | 2887 | 30860 | 320 | 1348 | 34.7 | 178 | 0.10 |
| 2019 | Hysterectomy | 3083 | 19077 | 271 | 1312 | 33.6 | 178 | 0.10 |
| 2019 | Spinal fusion | 2313 | 66112 | 1063 | 1360 | 39.5 | 176 | 0.16 |
| 2020 | Appendectomy | 2065 | 18543 | 408 | 1592 | 45.5 | 177 | 0.09 |
| 2020 | TKR | 3521 | 30092 | 277 | 1107 | 28.8 | 178 | 0.08 |
| 2020 | THR | 2500 | 29793 | 322 | 1347 | 39.4 | 178 | 0.15 |
| 2020 | Hysterectomy | 2665 | 20219 | 317 | 1451 | 38.8 | 178 | 0.08 |
| 2020 | Spinal fusion | 2125 | 68559 | 1188 | 1497 | 44.6 | 176 | 0.21 |
| 2021 | Appendectomy | 2008 | 17084 | 431 | 1477 | 43.8 | 177 | 0.07 |
| 2021 | TKR | 3986 | 28220 | 669 | 1129 | 27.1 | 178 | 0.06 |
| 2021 | THR | 2897 | 28018 | 773 | 1312 | 35.3 | 178 | 0.08 |
| 2021 | Hysterectomy | 2911 | 18578 | 265 | 1377 | 35.4 | 179 | 0.08 |
| 2021 | Spinal fusion | 2213 | 64560 | 1112 | 1423 | 42.4 | 176 | 0.18 |
| 2022 | Appendectomy | 691 | 17492 | 821 | 1344 | 72.8 | 177 | 0.18 |
| 2022 | TKR | 1592 | 24686 | 380 | 1106 | 42.6 | 179 | 0.09 |
| 2022 | THR | 1050 | 25172 | 500 | 1360 | 58.6 | 178 | 0.16 |
| 2022 | Hysterectomy | 1036 | 16860 | 440 | 1358 | 56.9 | 179 | 0.09 |
| 2022 | Spinal fusion | 777 | 58751 | 1899 | 1511 | 73.6 | 176 | 0.23 |

Abbreviations: SE, standard error; OOP, out-of-pocket cost; HFD, hospital-free days; TKR, Total Knee Replacement; THR, Total Hip Replacement.

**Appendix Figure 1. Cost and clinical outcomes by place of service and procedure type, 2018-2022**.

**Allowed Amount**

**Out-of-Pocket Costs**

**Hospital-Free Days**


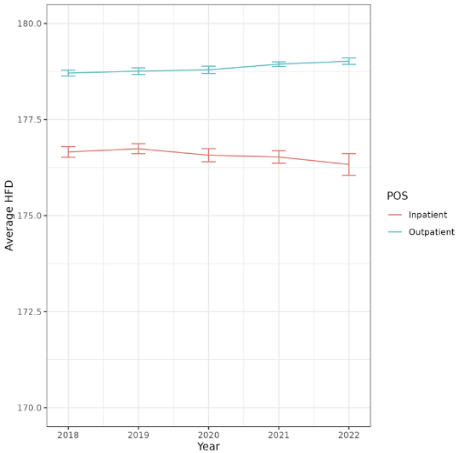

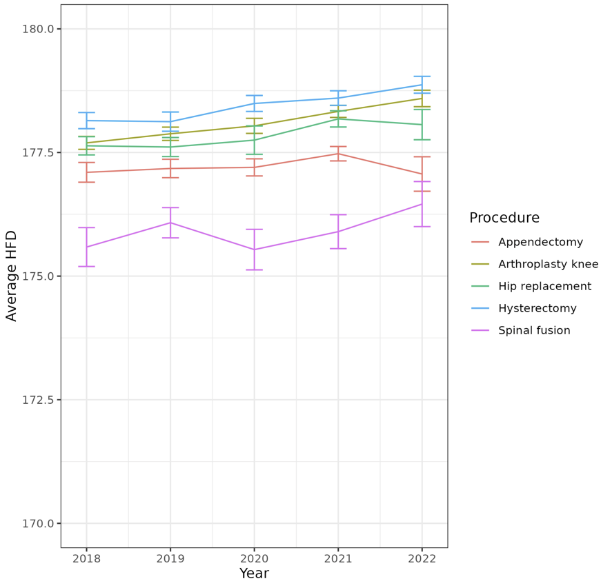

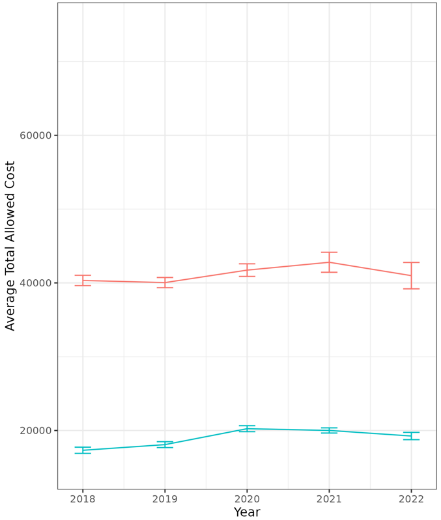

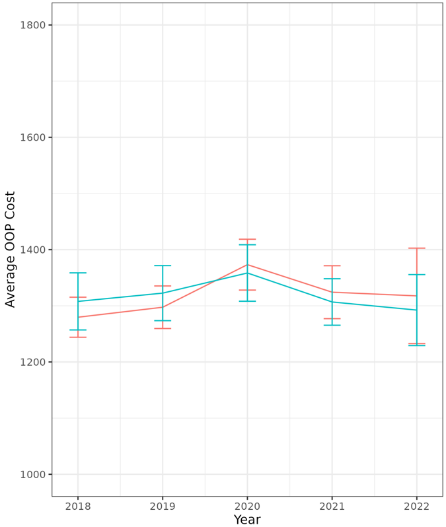

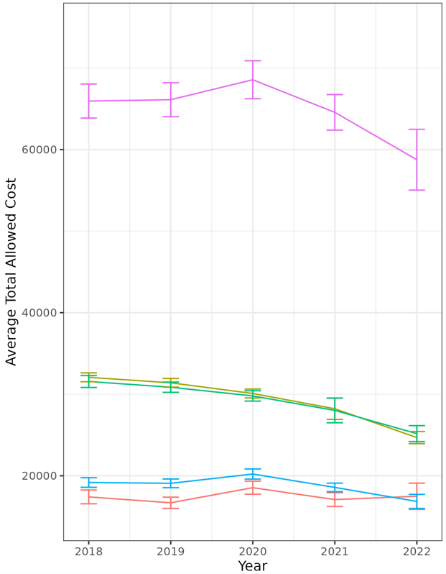

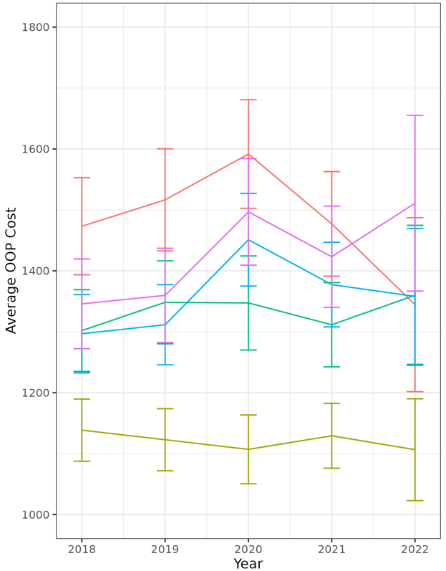


**Grouped by procedure type**

**Grouped by place of service**

Note: Data include 60,878 procedures across five Clinical Classifications Software (CCS) procedure groups with the greatest change in place of service from the week of January 1, 2018, to the week of May 14, 2022. The total allowed amount was calculated by aggregating allowed amounts on all claims within a two-week period following a patient’s surgery date. Out-of-pocket (OOP) costs include the sum of all copays, coinsurance amounts, and deductibles on patient claims occurring two weeks after the surgery date. Hospital-free days (HFDs) denote the count of days (out of 180) during which a patient was alive, was not admitted, and did not visit the emergency department within 6 months post-surgery. POS = place of service.

**Appendix Figure 2. Ratio of Cost and Clinical Outcomes by Median Income, % Black, and Labor Force Participation at the Census Tract Level: Ratio of 4^th^ vs. 1^st^ quartiles**


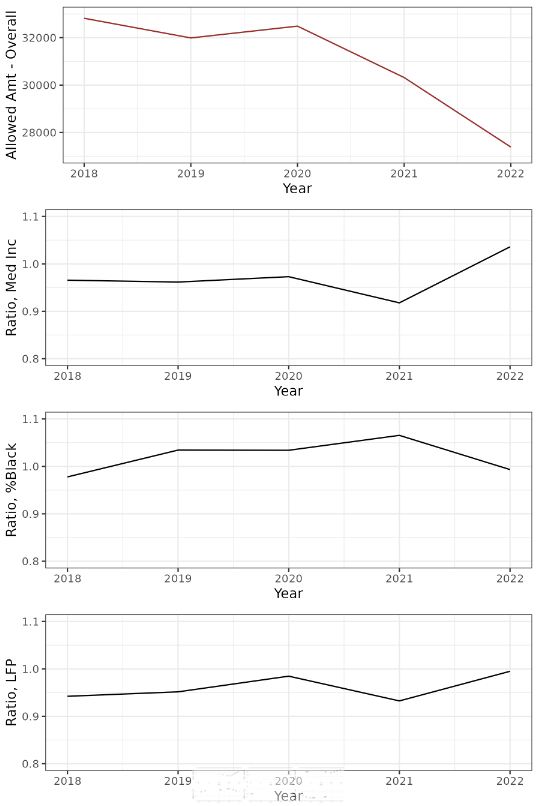

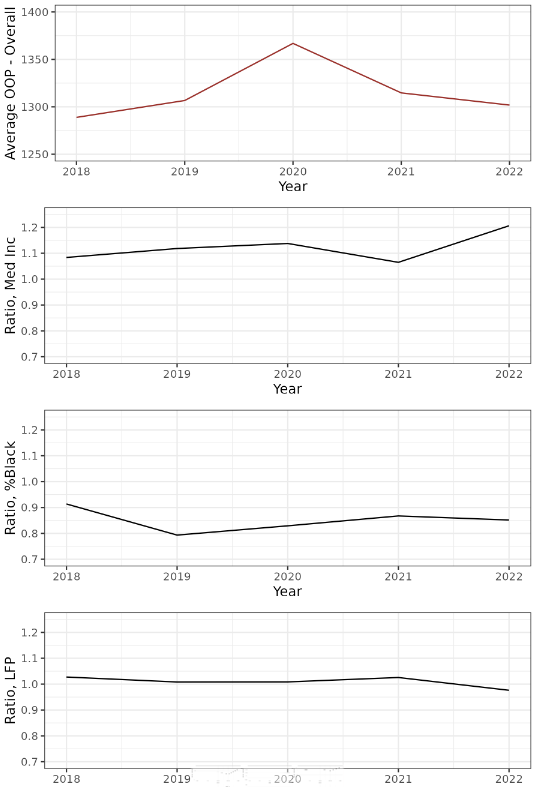

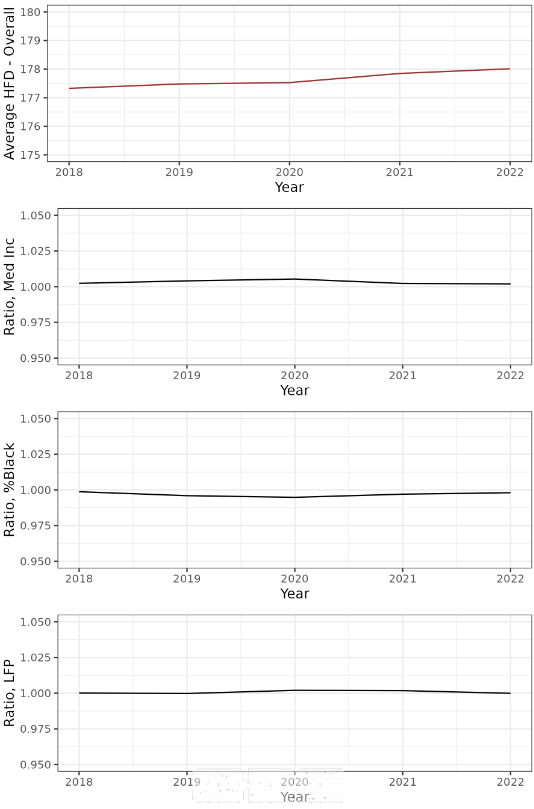


**Out-of-Pocket Costs**

**Allowed Amount**

**Hospital-Free Days**
